# Neural Patterns Reveal Lateral Occipital Complex Representation of Ensemble Mean Orientation

**DOI:** 10.1523/ENEURO.0137-26.2026

**Published:** 2026-06-30

**Authors:** Noam Khayat, Merav Ahissar, Shaul Hochstein

**Affiliations:** ^1^ELSC Safra Center for Brain Sciences, Hebrew University, Jerusalem 9190401, Israel; ^2^The Alexander Silberman Institute of Life Sciences, The Hebrew University of Jerusalem, Jerusalem 9190401, Israel; ^3^Department of Psychology, The Hebrew University of Jerusalem, Jerusalem 9190501, Israel

**Keywords:** ensemble perception, fMRI, LOC, perception, summary statistics, vision

## Abstract

Ensemble perception refers to the visual system's ability to extract summary statistics of groups of similar elements, manifested, for example, as efficient perceptual averaging. While this phenomenon has been extensively studied and characterized behaviorally, its underlying neural mechanisms remain largely unexplored. We ask if average orientation is represented in early visual cortex, known for sensitivity to local orientation, or higher cortical levels, including object- and scene-selective regions. Kahneman et al. (1992) classically proposed that object features are represented in “object files” and Ayzenberg and Behrmann (2022a) concluded that ventral representations form basis sets of local object features. Following these suggestions, we hypothesize that ensemble statistical characteristics are represented in similar “ensemble files.” This similarity leads to the possibility that ensembles may be represented in object-selective regions, specifically lateral occipital complex, LOC. Using fMRI, we studied neural representations of ensemble averages derived from bars with various orientations. Participants (8 males; 17 females) estimated their average orientation and occasionally reported this in a two-alternative forced choice task. We found modest, broadly tuned representations of average orientation in early visual area V3 and parietal regions. However, the strongest contribution emerged in the LOC, which manifested distinct voxel activity patterns reliably differentiating ensemble orientation averages. Multivoxel pattern analysis pinpointed LOC in both region of interest-based and searchlight-based decoding analyses and complementary correlation analyses. Notably, LOC decoding strength was correlated with participants' performance, suggesting functional importance of LOC representation. We conjecture that object-related LOC may represent ensemble characteristics in “ensemble files” similar to previously suggested “object files.”

## Significance Statement

Using fMRI, we find that object-related lateral occipital complex (LOC) has the major representation of average orientation of visual-bar ensembles. Representation strength correlated with participants' performance estimating this average, supporting its behavioral relevance. This neural observation supports classical hypotheses that object features are represented as “object files” and subsequent suggestions that features are represented at high levels of the ventral stream. We now observe that LOC representation of object features extends to ensemble characteristics. This novel finding informs our view of how the brain treats ensembles and also our understanding of what, for the brain, is an object.

## Introduction

Accumulating studies have shown that one strategy employed by the perceptual system is to rapidly summarize the basic structures of groups of similar items by computing their statistical properties, a process known as ensemble perception. The main perceived characteristic is ensemble mean, integrating local elements to represent global scenes. Such integration is extremely efficient and informative, forming an immediate first hypothesis of the gist of an object or scene. A principal enigma of ensemble perception is the finding that observers report precise perception of the ensemble mean without accurate knowledge of individual element parameters. How is the average computed without its constituents?

One resolution is suggested by reverse hierarchy theory (RHT), which divides between rapid vision at a glance for perception of the gist of the scene and slower vision with scrutiny, acquiring scene details (Ahissar and Hochstein, 1997, 2004; Hochstein and Ahissar, 2002; Hochstein, 2020). RHT proposes that scene gist is quickly represented at higher or intermediate cortical levels, which are the first accessible to conscious perception, whereas perceiving scene details requires slower return to lower cortical level areas. Indeed, theoretical accounts propose that ensemble perception may reflect a visual system feedforward mechanism, providing a stable, low-noise summary of sensory input supporting perceptual organization and guiding subsequent focused attention (Alvarez, 2011; Whitney and Yamanashi Leib, 2018; Corbett et al., 2023).

In a classic theoretical account of object perception, Kahneman et al. (1992) suggested that when an object is perceived, its basic features are also represented in an “object file.” Recently, Ayzenberg and Behrmann (2022a) presented evidence that the ventral visual pathway, at both single-unit and population-activity levels, exhibits greater sensitivity to local features than to complete shapes. They concluded that such a set of local features may be sufficient to recognize familiar objects that we encounter in day-to-day life. Albeit, these may be insufficient in more difficult situations, such as when learning new objects or encountering objects in novel contexts or encountering several objects that need to be identified, when input from dorsal pathway representations of global shape are required (Xu and Chun, 2009; Ayzenberg and Behrmann, 2022b). Taken together, these reports suggest that cortical representations of objects, specifically in the ventral visual pathway, may essentially include lists or files of features or characteristics of the viewed object.

Building on this framework, we hypothesize that ensemble summary representations may also similarly contain a file of ensemble features and integrate ensemble elements into a single object-like unit. Such integration may be supported by higher-level visual regions involved in object perception. We now test the novel proposal that this integration may be supported by a higher-level visual region, specifically one involved in object perception. A key candidate for such representation is the lateral occipital complex (LOC; Malach et al., 1995; Grill-Spector et al., 1998; Kourtzi and Kanwisher, 2001; Ayzenberg and Behrmann, 2022a), the focus of the current study.

Several recent fMRI studies asked where ensemble statistics are represented in the visual hierarchy. Tark et al. (2021) used ensembles of oriented Gabor patches and found ensemble representations in both early (V1–V3) and higher-level frontoparietal visual areas. Reconstructing the average orientation from voxel activity patterns, they demonstrated summary orientation information represented across these regions. However, they did not study high-level regions along the ventral visual pathway, such as scene-selective parahippocampal place area (PPA) or object-selective LOC, which, as outlined above, may be primary in global gist perception.

Using fMRI adaptation, studies by Cant and Xu (2012; 2015; 2017) found that the PPA responds selectively to ensemble features, whereas the LOC is more selective to individual objects within the ensemble. They presented image sequences, primarily ensembles of high-level real-world objects (e.g., fruits, instruments) and surface textures, and assessed the degree of adaptation despite individual image variation. While PPA remained adapted across sequences of different images of the same ensemble, LOC exhibited release from adaptation in this condition. However, these studies did not use ensembles of basic low-level elements but rather scene-like displays of grouped real-world objects, which may have contributed to engagement of scene-selective regions such as PPA (Epstein and Kanwisher, 1998; Xu and Chun, 2007, 2009), and localized LOC responses to the constitutive elements, overshadowing responses to the ensemble as a whole.

While these previous findings are important, they leave open essential issues regarding ensemble representation. We now use oriented bars to minimize object-like elements while systematically examining ensemble representations across the range of visual regions implicated by previous work, including early orientation processing visual areas (V1–V3) and scene-related processing areas (e.g., PPA), and focusing on object-processing area, LOC. Our stimuli were chosen to be simple enough not to be seen as objects themselves. Using ensembles of oriented bars, stimuli which are typically represented individually in the early visual cortex (EVC; Kamitani and Tong, 2005), we asked whether early visual areas also represent the ensemble-level information, such as average orientation, or whether such computations are deferred to higher cortical areas known to integrate information for complex visual representations. We therefore examined ensemble representations across multiple levels of the visual hierarchy, focusing on explicit ensemble perception, which is expected to yield robust neural responses (Harel et al., 2014). Ensemble-level coding in the object-selective cortex, such as the LOC, would support the novel notion that ensembles are treated as unified perceptual entities, characterized by their features and represented like objects by their “ensemble files.” In addition, finding ensemble representation in LOC would enhance our understanding of the role played by LOC itself in object perception.

## Materials and Methods

### Participants

Twenty-five healthy volunteers (students and staff from the Hebrew University of Jerusalem; mean age, 26.5 years; 17 females) participated in the study and completed all fMRI scans of our experiment. One additional participant was excluded due to excessive head motion. The methodology and experimental procedures for this study were approved by the Human Research Ethics Committee of the Hebrew University and the Helsinki Ethics Committee of Hadassah Hospital. Written informed consent was obtained from each participant before the procedure. All remaining participants were included in the primary analyses. To verify that results cannot be attributed to poor behavioral performance of a few participants, additional control multivoxel pattern analyses (MVPA) were conducted excluding the lowest-performing participants (see ROI-based MVPA section below).

### Apparatus and experimental setup

Visual stimuli were presented on an MR-compatible LCD screen (resolution, 1,920 × 1,800; refresh rate, 60 Hz; NNL LCD Monitor, NordicNeuroLab) located behind the scanner. The screen was made visible to participants via a tilted mirror attached to the head coil. Participants responded using an MRI-compatible eight-button response box (Current Designs). Auditory feedback was given via MRI-compatible insert earphones (Sensimetrics S14). The experiment was implemented using Python interpreter version 3.8 and the PsychoPy library (Peirce et al., 2019).

### Main fMRI experiment: stimuli

The central aim of our study was to find where in the brain are visual stimulus ensembles represented. We used ensembles of differently oriented bars, presented simultaneously, and an explicit perception paradigm, to maximize expected ensemble representation signal.

**Figure 1.**
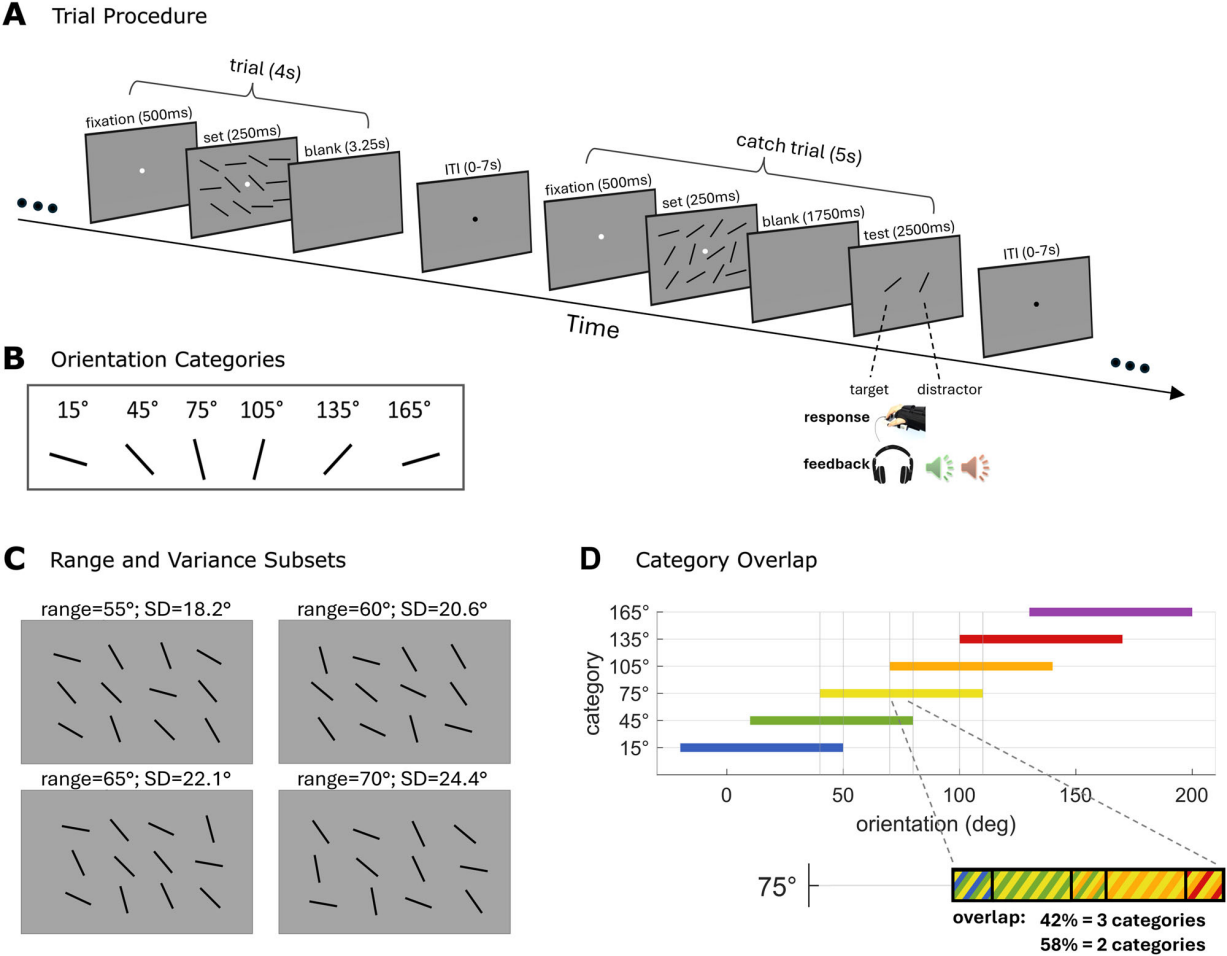
Experimental paradigm and stimuli. ***A***, illustration of experimental procedure with a standard nontest trial, and a catch trial with a 2-AFC mean estimation task following ensemble presentation. In all cases, participants were asked to explicitly estimate the mean orientation and use this estimation behaviorally in the catch trials. ***B***, Six mean-ensemble orientation categories of the experiment. ***C***, Examples of four range and variance subsets for a category of 45°. Though they are all of the same orientation category, they differ in the variability, jitter, and combinations of positions of the individual bars over the grid. ***D***, Illustration of maximal category range overlap. For the category of mean orientation 75° (yellow), all orientations are present in at least one more category; vertical lines delineate orientation ranges that belong to three categories. Zoom-in at the bottom illustrates the segments of these categories which overlap with different categories, as represented by the corresponding two or three colored lines.

Each trial presented an ensemble of 12 black bars with six different orientations, each repeated twice (Fig. 1*A*). Each bar was ∼1.59 × 0.07° of the visual angle. There were six categories, corresponding to mean orientations spaced 30° apart, ranging from 15 to 165° (0°, horizontal; 90°, vertical; Fig. 1*B*). To introduce variability within each category, several modifications were made to the original 3 × 4 grid layout (rows × columns). First, a local jitter of up to 17 pixels was applied to each individual bar, and a global jitter of up to 40 pixels was applied to the entire array, both vertically and horizontally. We also created four fixed orientation subsets per category, each defined by a distinct orientation range and variance (Fig. 1*C*). On each trial, one of these subsets was used to generate the ensemble. For each category subset, bars were arranged using two different spatial configurations on the grid. For the MVPA analysis, trials were collapsed across variability levels, as variability was not treated as a separate experimental factor.

To reduce reliance of behavioral responses on individual stimulus bars, we assured that each bar could belong to more than one ensemble category. We used wide orientation ranges within each ensemble leading to substantial overlap in orientation between categories: 45–57% overlap between neighboring categories (i.e., those 30° apart), depending on the specific subset range (corresponding to 25–40° of orientation overlap). As a result, for each category, 91–100% of the orientation range overlapped with neighboring categories. Furthermore, depending on the ensemble orientation range, categories differing by 60° could also share orientations, particularly at the center and edges of the range. Such overlap allowed each individual bar to plausibly belong to two or three categories: 23–42% of the orientation range overlapped across three categories, and the rest overlapped across two (see example in Fig. 1*D*). This overlap reduced the possibility that any single bar orientation could cue the ensemble category, thereby discouraging item-based strategies and promoting ensemble-level integration.

To further minimize the use of a subsampling strategy—where observers might attend to just one or two elements instead of integrating across the ensemble (Myczek and Simons, 2008; Maule and Franklin, 2016)—we limited stimulus duration to 250 ms and presented a fixation dot before each trial for central fixation. Bar positions varied unpredictably across trials, making it difficult to anticipate or selectively focus on any specific item. Together, these design features limit the feasibility of subsampling or saccadic selection, promoting global ensemble processing.

### Experimental procedure

We used a fast event-related design, with short trials implementing an explicit orientation-averaging task. Each trial began with a fixation dot presented for 500 ms, followed by an ensemble of oriented bars shown for 250 ms and then a blank screen for 3.5 s. Participants were instructed to “catch” or “perceive” the mean orientation of the ensemble. To maintain engagement and motivation, 20% of the trials in each run were catch trials, randomly interleaved with standard trials. Each of these trials included a two-alternative forced-choice (2AFC) test at its end: after a 1.75 s blank screen, two test bars appeared side by side, and participants selected which bar was closer to the perceived mean orientation by pressing a left or right (1 and 2) button. Auditory feedback was provided via earphones. The total duration of a catch trial was 5 s total (500 ms fixation, 250 ms ensemble stimuli, 1.75 s blank and 2.5 s test and response window), while standard (nontest) trials lasted 4 s. In nontest trials (80% of trials), the blank screen was followed by the reappearance of the fixation dot, signaling the absence of a test and preparation for the next trial.

Participants were instructed to maintain central fixation throughout and to avoid eye movements. To prevent participants from using simple outlier rejection strategies (Khayat and Hochstein, 2018), both test bars were always selected from within the range of orientations presented in the ensemble on that trial. Test bar relative similarity made the task difficult, encouraging close attention to ensemble averages. This design increased task difficulty by eliminating salient range-based cues, so that performance depended only on the precision of trial-specific mean estimation.

Prior to the MRI session, all participants completed one practice run of 60 trials, including 12 catch trials, outside the scanner to familiarize them with the task.

The main task included seven runs of 60 trials each, totaling 420 trials per participant. Runs were randomly ordered across participants. Of these, 84 catch trials (20%) were excluded from the fMRI analysis to avoid potential confounds from explicit individual-item perception. The remaining 336 trials were used for MVPA. Catch trials were included only for behavioral assessment of accuracy and reaction time (RT).

### Behavioral data analysis

In the behavioral tests of catch trials, mean estimation accuracy and response time (RT) were collected and analyzed. Trials with no response were excluded from analysis. Accuracy rates were computed as a function of Δ, the relative distance of test items from the ensemble mean (Δ = |distractor − mean| − |target − mean|), and distance of target from actual mean (0, 5, or 10°). Paired *t* test and effect size using Cohen's *d* were performed to assess the behavioral effect of ensemble perception.

### MRI scanning parameters

The blood oxygenation level-dependent (BOLD) fMRI measurements were acquired using a 3 Tesla Magnetom Skyra Siemens scanner and a 32-channel head coil. The fMRI protocols were based on a multiband echoplanar imaging sequence with the following parameters: TR, 1 s; TE, 30 ms; flip angle, 68°; acquisition matrix, 95 × 95; FOV, 240 × 240 mm; and multiband factor, 4. A total of 36 slices with 2.5 mm slice thickness (with no gap) were oriented in an oblique position covering the whole brain, with functional voxels of 2.5 × 2.5 × 2.5 mm. In addition, high-resolution T1-weighted magnetization-prepared rapid acquisition gradient-echo images were acquired (1 × 1 × 1 mm resolution).

### MRI data processing

Data analysis was conducted using MATLAB (version R2023a; The MathWorks) along with several MATLAB-based toolboxes, including Statistical Parametric Mapping (SPM12; Brett et al., 2002; Friston, 2003), The Decoding Toolbox (TDT; Hebart et al., 2015), and NeuroElf v1.1. In addition, BrainVoyager QX (Version 22.2; Brain Innovation) was used for surface-based retinotopy analysis and visualization.

SPM was used for preprocessing and for computing the general linear model (GLM) for both the main experiment and the functional localizer runs. TDT was used for MVPA of the main experiment data. NeuroElf was used for cortical surface inflation and surface-based visualization.

Preprocessing was performed using SPM and included head motion correction, coregistration to the anatomical image, normalization to the standard anatomic Montreal Neurological Institute (MNI) template space (using the original voxel resolution of 2.5 mm isotropic voxels), and high-pass filtering (cutoff, two cycles per run). Spatial smoothing with an 8 mm full-width at half-maxima Gaussian kernel was applied only to the localizer and retinotopy data; MVPA data remained unsmoothed to preserve fine-grained voxel patterns.

The cortical surface was reconstructed from each participant's anatomical scan and transformed into standard MNI space. BOLD responses were modeled using a conventional GLM approach, with regressors constructed by convolving stimulus onset times with a double-gamma hemodynamic response function.

### Region of interest (ROI) selection

**Figure 2.**
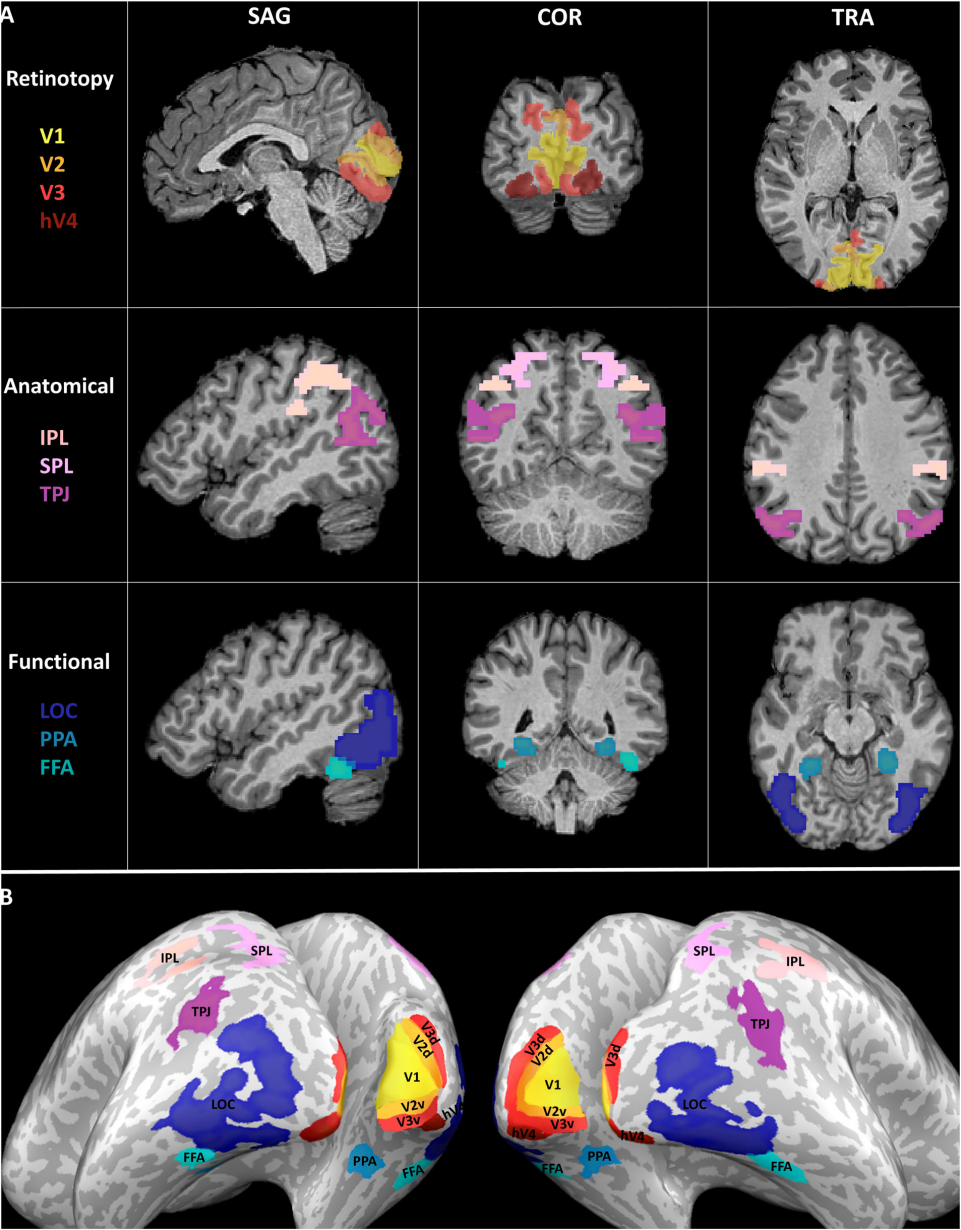
Example ROIs from a single participant overlaid on his brain. All 10 ROIs defined for one participant are shown. ***A***, ROIs overlaid on the T1-weighted anatomical image in sagittal (SAG), coronal (COR), and transverse (TRA) planes. ROIs are grouped by definition method—retinotopic, anatomical, or functional—and presented in separate rows. ***B***, The same 10 ROIs of this participant displayed on inflated cortical surfaces of both hemispheres from two viewpoints, illustrating lateral and medial surfaces. Details of coordinates (center of mass) and size (group mean ± SD) of ROIs are provided in Extended Data Figures 2-1 and 2-2, respectively.

ROIs were selected a priori to sample multiple levels of the visual processing hierarchy and to test alternative accounts of ensemble representation. Ten bilateral ROIs were selected based on their association with relevant features related to ensemble perception. These comprised early visual areas (V1, V2, V3, and hV4), high-level visual category-selective regions [LOC and fusiform face area (FFA), PPA], and anatomically defined parietal regions [inferior parietal lobule (IPL), superior parietal lobule (SPL), and temporoparietal junction (TPJ)]. These were volume-based ROIs with 2.5 mm isotropic voxel resolution restricted to gray matter. Some ROIs were previously associated with ensemble perception, including PPA (Cant and Xu, 2012, 2015, 2017), SPL, and EVC regions (Tark et al., 2021). Other regions were selected based on their involvement in related perceptual and attentional processes relevant to ensemble perception, including object and scene perception, orientation coding, integration of visual features, spatial attention, and gestalt organization (Malach et al., 1995; Epstein and Kanwisher, 1998; Wilkinson et al., 2000; Corbetta and Shulman, 2002; Giesbrecht et al., 2003; Kamitani and Tong, 2005; Huberle and Karnath, 2012). ROIs were defined individually for each of the 25 participants. Figure 2 shows the complete set of ROIs from one participant, overlaid on the three anatomical planes of the T1-weighted scan and on an inflated cortical surface for enhanced visualization.

10.1523/ENEURO.0137-26.2026.f2-1 Figure 2-1 MNI coordinates per ROI. Details of ROI coordinates (center of mass). Download Figure 2-1, DOCX file.

10.1523/ENEURO.0137-26.2026.f2-2 Figure 2-2 **ROI size (voxel count).** Details of ROI size (group mean ± SD) Download Figure 2-2, DOCX file.

Category-selective regions were defined using independent functional localizer scans, and early visual areas were defined using retinotopic mapping, as described below.

Four visual retinotopic areas (V1, V2, V3, and hV4) were identified based on retinotopic mapping. Using a phase-encoding approach (Sereno et al., 1995; Engel et al., 1997), we constructed individual polar maps and overlaid them on inflated cortical surfaces (as implemented by the BrainVoyager QX software). The retinotopic borders were delineated manually (Wandell et al., 2007) in each hemisphere where the phase reverses, as illustrated with the phase color. Volume-based ROIs for these areas in both hemispheres were then extracted. A false discovery rate (FDR) criterion was applied to correct for multiple comparisons.

To anatomically define the parietal regions—IPL, SPL, and TPJ—we used the WFU PickAtlas toolbox for MATLAB (Maldjian et al., 2003). Details of coordinates (center of mass) and size (group mean ± SD) of ROIs are provided in Extended Data Figures 2-1 and 2-2, respectively.

### Localizer scans

In order to localize and define the 10 selected ROIs, we interleaved a few additional runs, as described below.

#### Retinotopy analysis scans

To map and define retinotopic regions, we conducted an additional fMRI scan using a rotating checkerboard wedge stimulus for polar angle mapping (Sereno et al., 1995; Engel et al., 1997). The stimulus consisted of a monochromatic checkerboard wedge that rotated clockwise around the fixation point, flickering at a counter-phasing frequency of 7.5 Hz to maximize visual cortex activation. The radial size of the checkerboard segments was adjusted to account for the cortical magnification factor. Each wedge spanned 45° of the visual angle and extended from the fixation point to 8.5° into the visual periphery.

Each scan comprised six full cycles of the rotating wedge, with each cycle lasting 24 s. Participants were instructed to maintain fixation on a central point. To ensure fixation was sustained, the fixation point changed color from red to green for 100 ms, nine times per scan, and participants were asked to count the number of color changes.

Phase-encoding analysis with cross-correlation was used to extract phase information. Borders between the regions were identified at phase reversals and drawn over the inflated brain surface to define the regions. Additional scans using expanding and contracting rings were performed and used to map visual field eccentricity and assist in delineating the borders between retinotopically defined ROIs.

#### Functional localizer scans

LOC, PPA, and FFA were identified using two functional localizer runs. LOC was identified in both hemispheres using the standard contrast of the following images: intact object > scrambled object [familywise error rate (FWE) < 0.05; Grill-Spector et al., 1999]. FFA was defined by the contrast face > house (FWE < 0.05; Kanwisher et al., 1997) and PPA by house > face (FWE < 0.05; Epstein and Kanwisher, 1998).

ROI size (voxel number) of LOC, PPA, and FFA naturally varied across participants (see group-level size and coordinates in Extended Data Fig. 2-2). LOC voxels overlapping with parahippocampal gyrus (PPA) were excluded to maintain LOC specificity. In the few cases of functional overlap with fusiform face-selective cortex (FFA), LOC was defined using a more selective contrast, contrasting intact objects with all three other conditions (scrambled objects, houses, and faces). All ROIs were subsequently matched in voxel number for MVPA analyses, ensuring comparability across participants.

#### Equalizing size of ROI set by selecting the 100 most responsive voxels

Since the ROI size can influence the results of ROI-based MVPA (Schreiber and Krekelberg, 2013; Walther et al., 2016), we created an ROI set with an equal number of voxels. We performed a univariate analysis with all average orientation conditions modeled together as a single regressor versus baseline of rest periods (while fixating on a blank screen). This yielded a single contrast map per participant (condition > fixation), from which we calculated voxel-wise *t* values, creating a *t* statistic map per participant. We then sorted and ranked the voxels of each ROI by responsiveness to bar stimuli and selected the 100 most active ones. The new set comprised the 10 ROIs in each of our 25 participants (a few individual ROIs included <100 voxels, so all voxels were taken for the analysis). These data of 100 voxels per ROI were used for MVPA. Importantly, the GLM of the univariate analysis did not differentiate between the different average orientation categories; rather it included all categories together as a single condition; thus, it avoided the possibility of circularity of selecting the voxels that hold information that can differentiate between the categories for MVPA.

Using the univariate analysis GLM, we also estimated each ROI's overall activation level by computing percent signal change (PSC) relative to fixation. For this, we extracted a stimulus condition > fixation contrast map, using the full ROI mask (rather than the top-100 subset). Because the contrast already represents the difference from baseline, the mean value of all voxels in the ROI served as an estimate of PSC. This allowed us to quantify overall activation and responsiveness to the oriented bar ensemble stimuli, independent of the specific mean orientation, across all ROIs (Extended Data Fig. 4-1).

### Decoding analysis

#### ROI-based statistical analysis of ensemble average orientation information

We used MVPA to assess whether ensemble average orientation information is present in distributed activity patterns across cortical regions. Classification was performed using a linear support vector machine (SVM) implemented via the library for SVM (Chang and Lin, 2011), as implemented in TDT (Hebart et al., 2015). This approach was used to assess the discriminability of activation patterns across mean orientation categories within each ROI.

Decoding performance within each ROI was assessed using a leave-one-run-out cross-validation approach. In each fold, the classifier was trained on data from all but one run and tested on the held-out run. All analyses excluded catch-trial data.

Statistical analysis was applied to the cross-validated decoding accuracy (%). In each ROI, we determined whether the accuracy (across participants) was significantly greater than the chance level using a permutation test. The condition labels were permuted across for each ROI in each participant. This procedure was repeated 1,000 times. The *p* values of the permutations test were corrected for multiple comparisons using Sidák correction.

#### Whole-brain searchlight MVPA

In addition to the predefined ROIs, whole-brain searchlight analysis was used to assess ensemble representations in regions not selected a priori, providing a complementary and unbiased assessment across the brain.

We applied a volume-based searchlight analysis (Kriegeskorte et al., 2006). For each participant, we searched iteratively through the brain using a cubic search window comprising 125 voxels (5 × 5 × 5; 12.5 × 12.5 × 12.5 mm^3^) for a multivoxel pattern that carries ensemble average orientation information. On each iteration, the window was centered on a new MNI voxel, and we performed a classification analysis similar to the standard ROI-based MVPA, with the classifier trained and tested on the discrimination between different ensemble average orientations.

Statistical analysis was conducted on the ensemble average orientation classification accuracy derived from the searchlight analysis. One-sample, two-tailed *t* tests were performed to identify search windows with significant classification estimates across 25 participants. The resulting *t* value statistical maps were corrected for multiple comparisons using a FDR threshold [*q*_(FDR)_ < 0.05].

For visualization, the significant searchlight clusters are shown alongside group-level ROIs exhibiting the highest ensemble average sensitivity. Group-level ROI maps were created by calculating, for each MNI voxel, the proportion of participants whose individual ROIs included that voxel and displaying voxels thresholded to include those shared by at least 20% of participants. Searchlight clusters are then compared with ROI-based MVPA finding results.

### Correlation-based multivoxel pattern similarity analysis

In addition, as a complementary approach to the classification analysis, we performed a correlation-based MVPA. While classification identifies discriminative information by weighting voxels according to their informativity, correlation handles all voxels equally and provides a more direct estimate of representational similarity. For each ROI and participant, we first split the data into two groups (“odd” and “even” runs). Voxel-wise beta estimates were normalized by subtracting the mean across conditions for each voxel (Haxby et al., 2001; Garrido et al., 2013), providing a measure of stimulus information represented by the patterns. We then averaged beta maps across odd and even runs separately, yielding two activation patterns per condition. The effects of stimulus differences between runs were canceled by using odd and even runs in the across-participant randomized presentation order. Correlating odd and even patterns across all six conditions produced a 6 × 6 correlation matrix per ROI, reflecting pairwise correlations between conditions.

Observation on these matrices enabled assessment of the between-category similarity structure, as reflected in the dependence of the correlation on orientation difference. In addition, we quantified the precision and strength of the mean stimulus information as the difference between the correlation for same-condition pairs (matrix diagonal) and the correlation for different-condition pairs (off-diagonal). To test statistical significance, participant-level same-minus-different values were compared against zero using two-tailed *t* tests, with *p* values corrected for multiple comparisons across ROIs.

### Behavior-decoding correlation analysis

Following the decoding analysis and determining the ROI of major ensemble representation, we assessed the relationship between behavioral performance and neural ensemble representation, by computing the correlation between individual participants' behavioral accuracy on catch trials and ROI-based decoding accuracy using Spearman's rank correlation coefficient. Since correlation estimates can be sensitive to extreme values in small samples (*n* = 25), extreme outliers were excluded on a per-ROI basis from both behavioral and MVPA data using a ±2 SD criterion; this affected at most 1–2 participants and only in a subset of ROIs. One participant with very low behavioral performance (32.5%) was excluded by the ±2 SD criterion; in addition, to confirm the robustness of the most significant correlation, data were reanalyzed also excluding a second participant with markedly below chance (40.9%) performance.

## Results

As described in the experimental procedure, participants viewed ensemble sets of bars with varying orientations on each trial and were instructed to perceive the average orientation. In 20% of the trials—randomly interspersed and without prior notice—a 2AFC task instructed participants to select which of two test bars best matched the perceived mean (Fig. 1*A*). To assess the neural encoding of ensemble information distinguishing the six average orientation categories, we used MVPA to decode category identity from voxel-wise beta activity patterns. A SVM classifier was trained and tested within predefined ROIs spanning early visual, parietal, and high-level category-selective areas, as well as a whole searchlight approach, allowing us to identify localized and distributed representations. In addition to classification accuracy, we evaluated the representational across-category structure of orientation ensembles through MVPA correlation-based similarity analysis. We also tested how neural patterns relate to behavioral performance.

### Behavioral performance in estimating the mean orientation

To assess participants' ability to extract average orientation from visual ensembles, we analyzed performance on catch trials using two behavioral measures: accuracy (percentage of correct responses) and RT. Each catch trial consisted of a 2AFC task that followed the simultaneous presentation of an ensemble of oriented bars, and participants were shown two test bars side-by-side and asked to select the one that best matched the average orientation of the preceding set.

**Figure 3.**
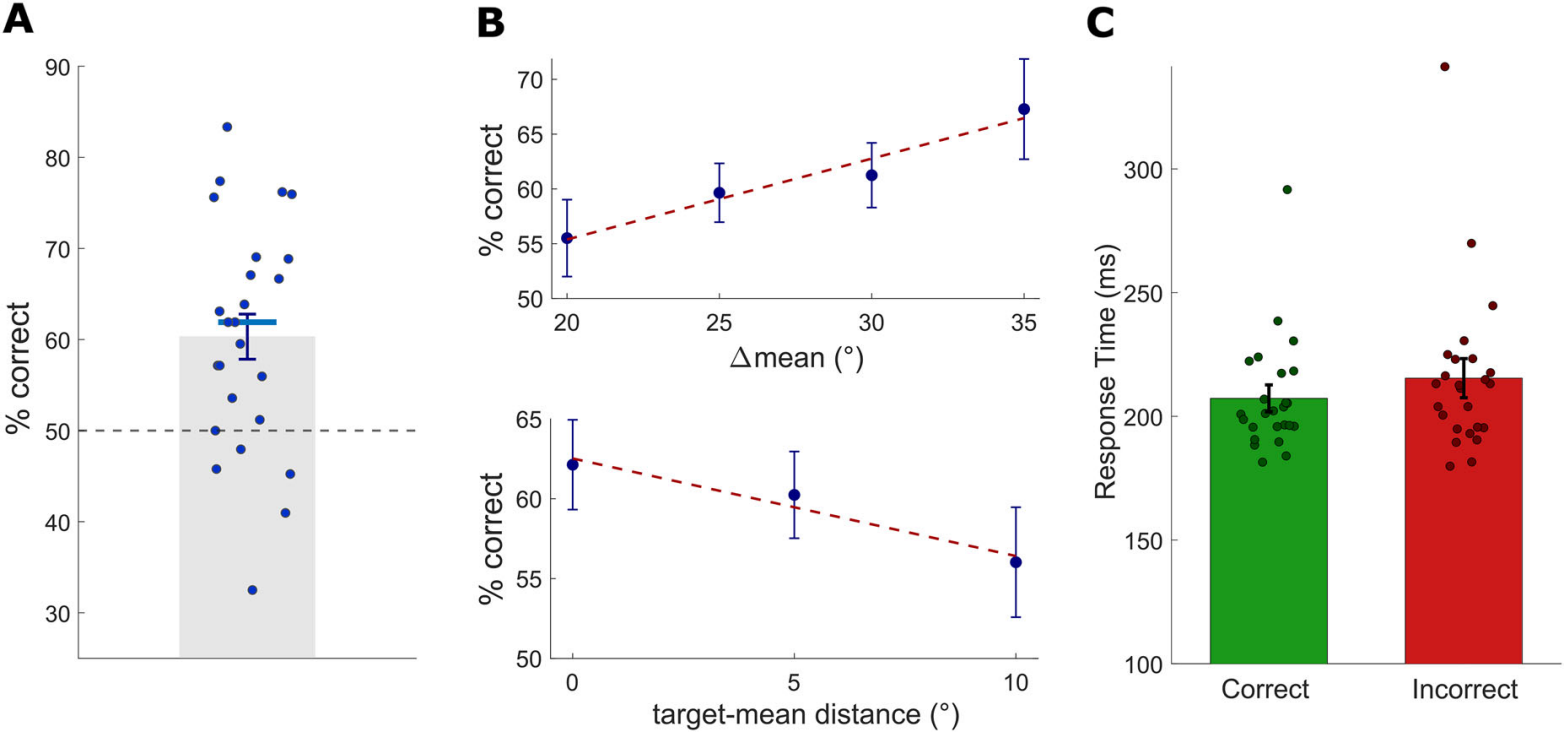
Behavioral performance in catch trials shows accurate perception of ensemble mean and dependence on test items. ***A***, Accuracy rates in catch trials per participant with mean and median values. The dashed line is the chance level of 50%. ***B***, Accuracy rate as a function of the relative distance of test items from ensemble mean (top) and distance of the target item from the ensemble mean (bottom). ***C***, Response time in catch trials for correct (green) and incorrect (red) responses. Each dot in ***A*** and ***C*** represents a single participant; error bars represent the SEM; bars in ***A*** and ***C*** represent the mean value across participants; horizontal bar in ***A*** represents the median value across participants.

Performance accuracy was significantly above chance (mean across participants, 60.3% ± 2.5 SEM; median = 61.9%; *p* < 0.001; Cohen's *d* = 0.82; Fig. 3*A*). Accuracy improved as a function of larger Δ, the relative distance of test items from the ensemble mean (Δ *=* |distractor − mean| − |target − mean|; Fig. 3*B*, top), and decreased as the distance of the target from the ensemble mean increased (Fig. 3*B*, bottom).

The mean RT across catch trials was similar for correct (207.2 ± 4.5 ms SEM) and incorrect (215.4 ± 6.5 ms SEM; *p* = 0.08) responses, indicating that performance was not driven by a speed–accuracy trade-off (Fig. 3*C*).

Together, these results confirm that participants were attending, perceiving, and remembering ensemble averages, able to extract mean orientation information from the ensembles with above-chance accuracy, and that performance was systematically influenced by the orientation properties of test bars relative to the ensemble mean.

### ROI-based MVPA classification

The primary aim of this study was to identify brain regions that encode information about the ensemble mean orientation. To this end, we assessed multivoxel activation patterns associated with each mean orientation category across the 10 predefined ROIs.

**Figure 4.**
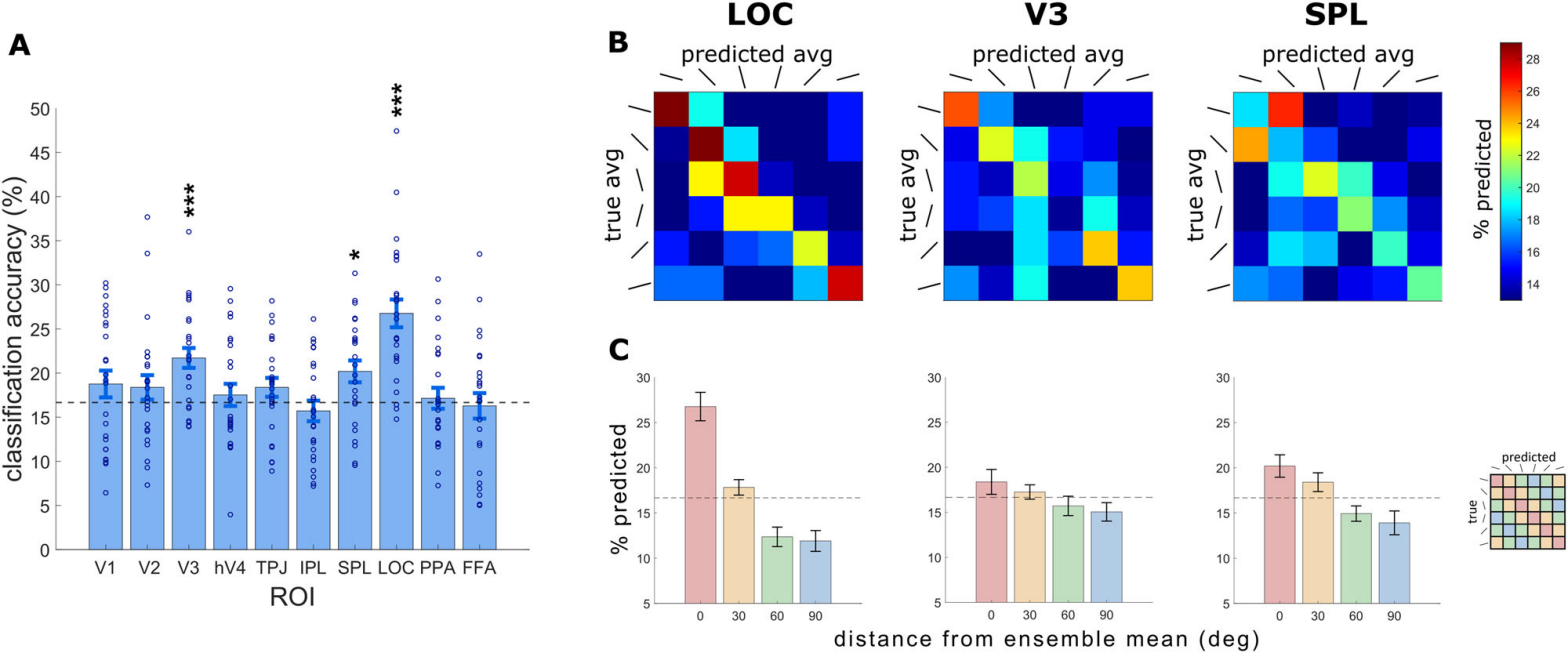
ROI-based MVPA decoding results. ***A***, classification accuracy per ROI, presenting the cross-subject mean values and SEM. The chance level is marked by the dashed horizontal line, and symbols represent individual participants. Highest accuracy is in LOC; significant accuracy also in V3 and SPL. ***B***, Group-level confusion matrices of ROIs with significant classification accuracy (LOC, *p* < 0.001; V3, *p* < 0.001; SPL, *p* < 0.05). Each square represents the proportion to which the true average stimulus is predicted by its voxel-pattern of activity as belonging to each of the six average orientations. Confusion matrices of classification accuracy for all ROIs are provided in Extended Data Figure 4-2. ***C***, The percentage of prediction as a function of distance from ensemble mean. Bar colors correspond to distance from the true mean, as illustrated on the distance matrix (right). Gradient decrease suggests between-category similarity representation. Group-level ROIs and univariate activation analyses are provided in Extended Data Figure 4-1 and the percentage of prediction as a function of angular distance from the true ensemble mean for all ROIs is provided in Extended Data Figure 4-3.

Figure 4*A* displays the group-level decoding accuracies for each ROI. Corresponding *p* values from two-tailed *t* tests and permutation testing, corrected for multiple comparisons (Sidák correction), are reported in Table 1. LOC exhibited the highest decoding accuracy for ensemble mean orientation (26.7%), significantly above the 16.6% chance level (*t* test *p* < 0.001, Sidák-corrected), indicating that it reliably encodes global average orientation. It outperformed all other regions, showing substantially better discrimination between mean orientation categories than V3 and SPL, which had the next highest decoding accuracies. V3 showed significant decoding (22%; *p* < 0.001, Sidák-corrected), supporting its involvement in ensemble processing. SPL also demonstrated above-chance performance (20%; *p* < 0.05, Sidák-corrected), though with lower accuracy.

10.1523/ENEURO.0137-26.2026.f4-1 Figure 4-1 **Group-level ROIs and univariate activation analyses. (A)** Group-level ROIs thresholded at ≥20% participant overlap. **(B)** group activation map illustrating overall activation for bar stimuli across all orientations. **(C)** Univariate analysis of mean percent signal change relative to fixation for each ROI. Overall activation was generally positive, but did not predict decoding performance, which relies on condition-specific spatial activation patterns rather than average signal strength. Bars represent group means; error bars indicate ± SEM. Download Figure 4-1, TIF file.

10.1523/ENEURO.0137-26.2026.f4-2 Figure 4-2 **Confusion matrices of classification accuracy for all ROIs.** Confusion matrices showing classification accuracy across all ensemble orientation mean categories. Values are averaged across participants. ROIs with significant classification accuracy are reported in the main text (Fig. 4B). Download Figure 4-2, TIF file.

10.1523/ENEURO.0137-26.2026.f4-3 Figure 4-3 **Percent of prediction as a function of angular distance from the true ensemble mean for all ROIs. Top:** Analysis scheme illustrating how confusion matrix entries were grouped by orientation difference. **Bottom:** Mean prediction rate at each distance, across ROIs. Bar colors correspond to distance from the true mean. Gradient decrease indicates similarity representation by decoding values that vary with distance from the actual mean orientation in several ROIs. Download Figure 4-3, TIF file.

**Table 1.** Statistical analysis of ensemble mean orientation decoding across ROIs

ROI	Two-tailed *t* test			Permutation test	
	*t*	*p*	*p(corr)*	*p*	*p(corr)*
V1	1.343	0.103	0.664	0.047	0.382
V2	1.286	0.114	0.702	0.076	0.546
V3	4.534	7 × 10^−5^	7 × 10^−4^	9 × 10^−4^	0.009
hV4	0.734	0.251	0.945	0.239	0.935
FFA	−0.500	0.294	0.969	0.653	1.000
PPA	0.248	0.428	0.996	0.338	0.984
LOC	6.452	6 × 10^−7^	6 × 10^−6^	9 × 10^−4^	0.009
IPL	−0.760	0.211	0.907	0.786	1.000
SPL	2.536	0.010	0.097	0.002	0.020
TPJ	2.052	0.029	0.257	0.066	0.495

Statistical results per ROI across 25 participants. Reported are *t* values and *p* values from two-tailed one-sample *t* tests, as well as permutation-based *p* values (*N* = 1,000), uncorrected and Sidák-corrected for multiple comparisons. Tests were performed on classification accuracy values against the chance level of 16.6%.

Since behavioral performance varied substantially and was poor for some participants, we repeated the MVPA while excluding (1) the two participants performing markedly below chance (32.5 and 40.9%) and (2) six additional participants performing near chance (45–53%). In both cases, decoding accuracy in LOC remained significant and comparable to the full-sample analysis (26.1% when excluding two participants; 27.7% when excluding eight participants), indicating that the primary findings were not driven by lower behavioral performance.

To further inspect classification performance, we visualized the group-averaged confusion matrices for all ten ROIs (Extended Data Fig. 4-2). The matrices for the three ROIs showing the strongest effects—LOC, V3, and SPL—are presented in Figure 4*B*, illustrating classification performance across all train-test category combinations. In these ROIs, higher values are concentrated along the main diagonal, indicating reliable discrimination between ensemble mean orientation categories (Fig. 4*B*).

To examine the structure of ensemble representations, we analyzed the distribution of classifier predictions as a function of angular distance from the true ensemble mean. For each ROI, we computed the percentage of predictions assigned to orientations located at 0, ±30, ±60, and ±90° from the actual mean. Since orientation is a circular feature, the maximum distance from the mean is 90°. Some regions, including LOC, SPL, and EVC, exhibited a clear gradient, with the highest proportion of predictions assigned to the correct mean and progressively lower proportions at increasing distances (Fig. 4*C*; Extended Data Fig. 4-3). This response profile suggests that ensemble mean orientations are not only decodable as discrete categories but may also be represented in a structured similarity space, in which neural activation patterns reflect the angular proximity between ensemble means.

In addition to the decoding analysis, we examined overall activation levels using PSC relative to fixation (Extended Data Fig. 4-1). The relation between univariate (PSC) and multivariate (MVPA) responses varied across regions (compare Fig. 4*A* and Extended Data Fig. 4-1). For example, retinotopic areas such as hV4 showed strong PSC but relatively low decoding performance, suggesting robust but nonensemble-selective visual responses to oriented bar stimuli. In contrast, higher-level regions like LOC exhibited moderate PSC but high decoding accuracy, consistent with more selective, pattern-based encoding of ensemble information. PSC confirmed stimulus-driven activation but showed no consistent relationship with decoding.

### Multivoxel pattern similarity

To further examine ensemble similarity representations, we conducted a correlation-based MVPA across orientation conditions. We tested the consistency of activation patterns within each orientation category, as well as similarity across categories differing in orientation, focusing on adjacent categories (±30°).

**Figure 5.**
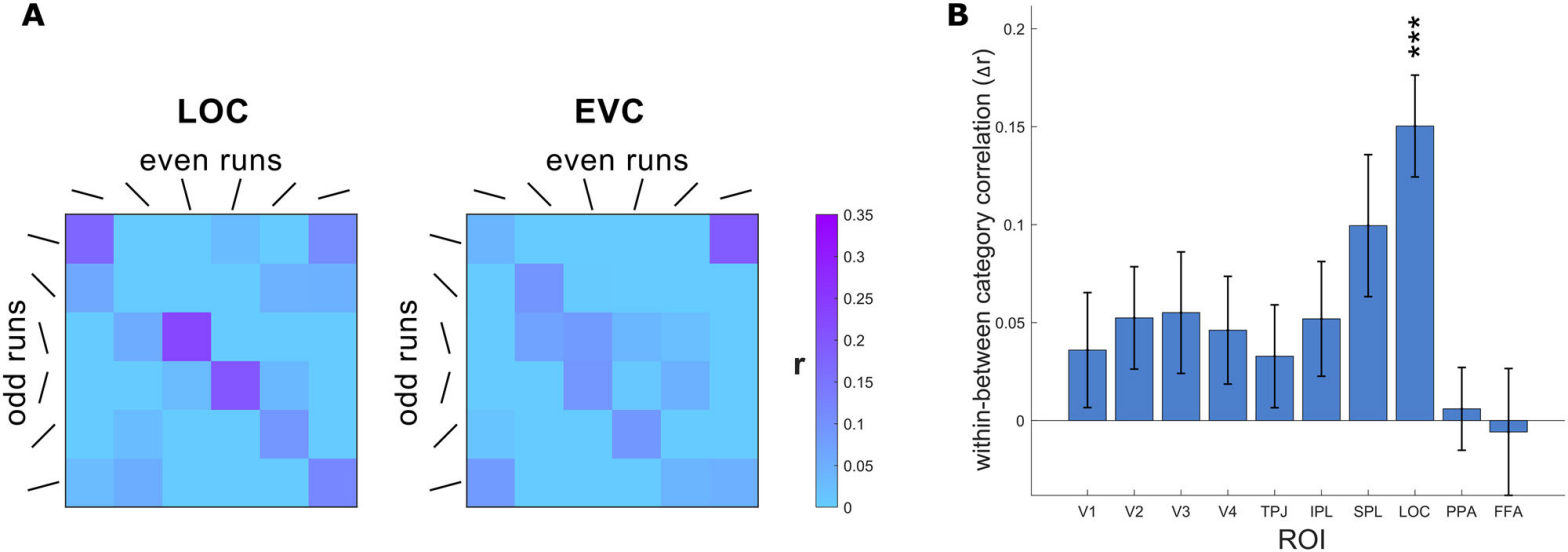
Correlation-based multivoxel pattern similarity. ***A***, Confusion matrices color transitioning represents Pearson’s *r* correlation values between odd runs and even runs, per combination of average orientation category, for LOC and averaged across EVC regions V1–V3. While LOC shows high correlation over the main diagonal, EVC shows a lower correlation on the main diagonal though higher correlation between adjacent categories. ***B***, Within-category minus between-category correlation difference for all ROIs. Correlation-based MVPA confusion matrices for all ROIs are provided in Extended Data Figure 5-1, and full statistical analysis of the correlation analysis of orientation ensemble mean is provided in Extended Data Figure 5-2.

Several ROIs, particularly EVC, V1–V3, showed higher correlations between adjacent categories, as visible in the correlation matrices (Fig. 5*A*; see Extended Data Fig. 5-1 for all ROI matrices). This pattern corresponds with the earlier results of decoding accuracy by the distance from the actual mean (Fig. 4*C*; Extended Data Fig. 4-3), supporting a similarity-based representation of mean orientation categories.

10.1523/ENEURO.0137-26.2026.f5-1 Figure 5-1 **Correlation-based MVPA confusion matrices for all ROIs.** Correlation matrices between odd and even runs for each pair of mean orientation categories, displayed separately for all 10 ROIs. Correlations along the main diagonal reflect within-category similarity, whereas off-diagonal bands indicate similarity between adjacent categories. Download Figure 5-1, TIF file.

10.1523/ENEURO.0137-26.2026.f5-2 Figure 5-2 **Correlation analysis of orientation ensemble mean.** Full statistical correlation analysis results. Download Figure 5-2, DOCX file.

In contrast, LOC showed stronger within-category correlations (main diagonal) and weaker correlations between adjacent categories, indicating a sharper representation of the mean orientation (Fig. 5*A*). This pattern was quantified by the within- minus between-category correlation measure, where LOC is the only region that reached a significant value (*p* < 0.001; Fig. 5*B*), supporting the conclusion that LOC encodes mean orientation with higher precision (see full statistical analysis results in Extended Data Fig. 5-2).

Together, these results suggest that high-level regions such as LOC may represent average orientations with narrower tuning curves and more precise mean representation, whereas early visual areas capture graded orientation similarities that mirror the stimulus structure.

### Searchlight-based MVPA classification

To confirm the ROI-based MVPA findings and identify additional cortical regions that may encode ensemble average orientation, we conducted a whole-brain searchlight analysis.

**Figure 6.**
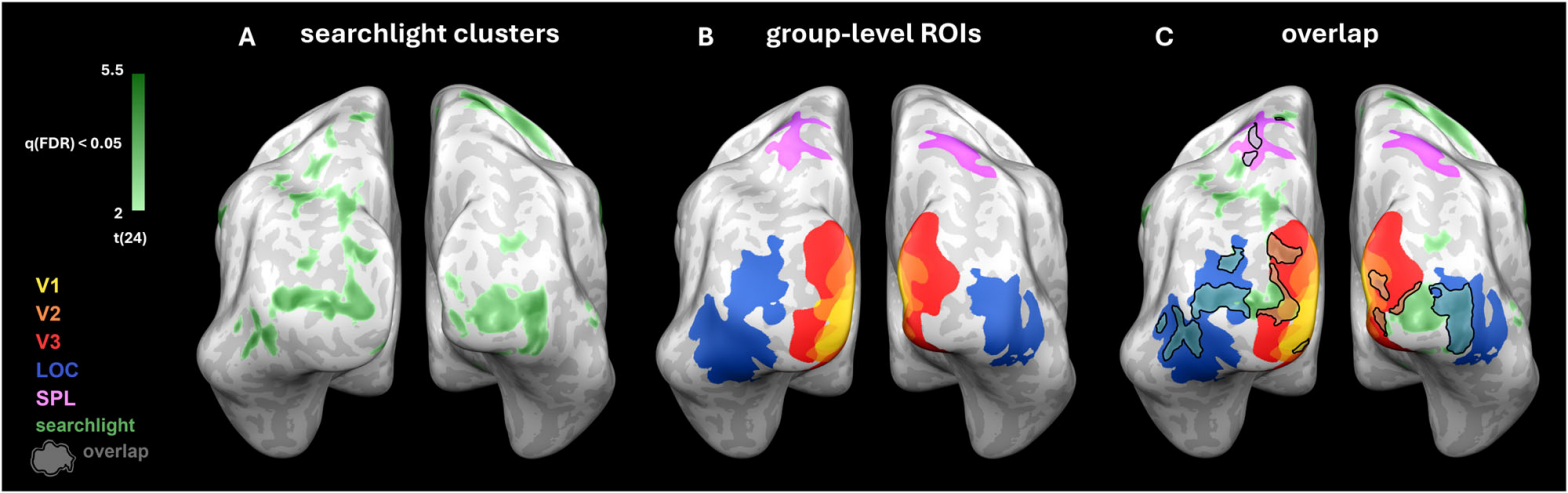
Searchlight-based MVPA over the whole brain. ***A***, significant clustered from the searchlight analysis [*q*_(FDR)_ < 0.05]. ***B***, Selected group-level ROIs—EVC, LOC, and SPL—thresholded at ≥20% participant overlap. ***C***, Overlay of searchlight significant clusters and group-level ROIs, with their overlap highlighted by a black solid line. Inflated surface of Colin 27 is used as a normalized template.

This analysis revealed several significant clusters with above-chance classification performance (Fig. 6*A*). Relevant bilateral group-level ROIs are displayed in Figure 6*B* and their overlap in Figure 6*C*, highlighted by black contours. The largest and most robust cluster was located in bilateral LOC, closely matching the group-defined LOC ROI. The peak coordinates of these clusters in standard MNI space corresponded well with the group mean LOC coordinates (left and right hemispheres: [−46, −70, −4] and [37, −102, 4], respectively). This spatial correspondence provides converging evidence with the ROI-based MVPA results, reinforcing the conclusion that LOC plays a central role in encoding ensemble mean orientation.

Additional loci beyond our ROIs emerged, most notably in the left intraparietal sulcus (IPS) (peak MNI coordinates: [−33, −70, 38]). Overlap was also observed in left SPL and EVC (V1–V3; Fig. 6*C*). Significant decoding in parietal and early visual areas suggests that ensemble representations may be distributed across multiple stages of the visual hierarchy with LOC appearing as a particularly prominent site of orientation mean information.

### Relationship between decoding accuracy and behavioral performance

Having found that LOC is the primary region in the MVPA analyses representing ensemble mean orientation, we next asked whether the strength of this representation correlates with behavioral performance on an individual-by-individual basis. Such a correlation would support the suggestion that the LOC representation is also functionally important. Accordingly, behavioral-decoding correlation was evaluated specifically for LOC.

**Figure 7.**
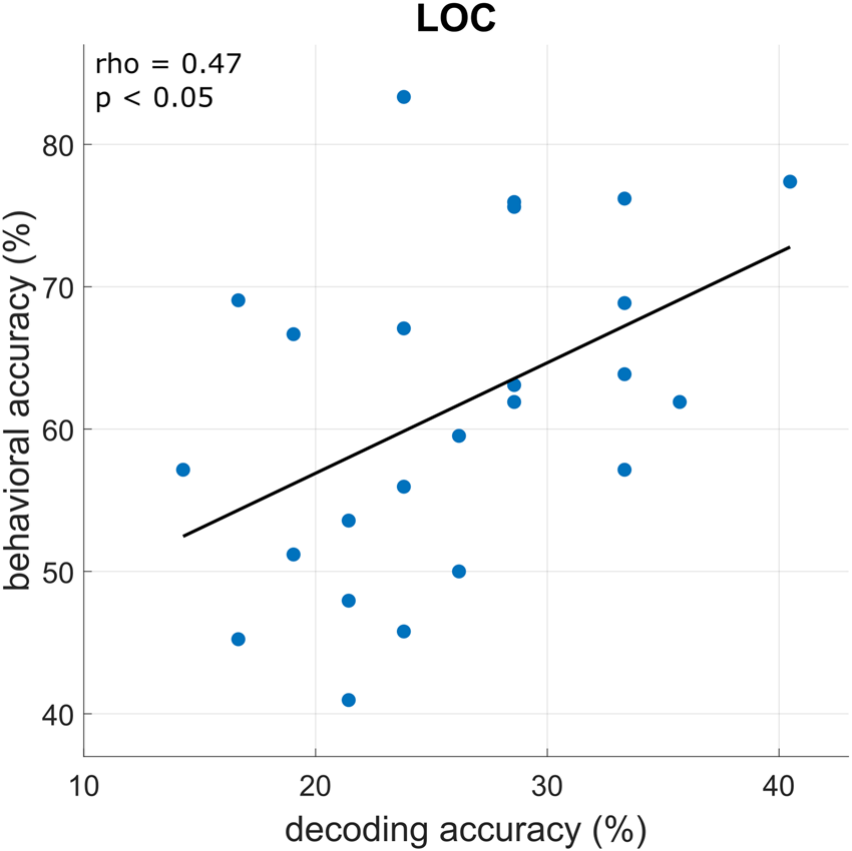
Correlation between neural decoding accuracy in LOC and behavioral catch-trial accuracy. Scatterplot shows the significant correlation between decoding accuracy in LOC and behavioral averaging accuracy across participants, indicating a link between neural decoding performance in LOC and behavioral averaging ability. Each dot represents one participant included after exclusion of one extreme outlier (±2 SD criterion), and the fitted regression line illustrates the significant positive correlation (Spearman *ρ* = 0.47; *p* = 0.02). Behavioral–neural correlations for all ROIs are provided in Extended Data Figure 7-1.

We computed Spearman correlation coefficients between decoding accuracy and behavioral accuracy across participants for LOC (*n* = 24, excluding one participant by the SD criterion, 32.5%) and found a significant correlation (*ρ* = 0.47; *p* = 0.02), illustrating that participants with more reliable neural discrimination of ensemble means in this region also tended to perform better in the behavioral task (Fig. 7). This relationship remained significant when excluding also the next lowest-performing participant (40.9%, not excluded by the predefined SD criterion, remaining with *n* = 23; *ρ* = 0.45; *p* = 0.03). This significant correlation suggests that voxel patterns in LOC reflect the averaging process demanded by the task.

10.1523/ENEURO.0137-26.2026.f7-1 Figure 7-1 **Behavioral–neural correlations for all ROIs.** Correlations between individual decoding accuracy and behavioral catch-trial accuracy across ROIs. Although some positive trends are observed, none reach Spearman correlation significance besides LOC, illustrating a relationship of the inter-individual variability in neural decoding and perceptual averaging behavioral. Each dot represents one participant included after exclusion of 2 extreme outliers (±2SD criterion applied to both behavioral and MVPA data), and the fitted regression line illustrates the linear slope. Download Figure 7-1, TIF file.

We also tested whether decoding accuracy correlated with individual participants' accuracy in the ensemble mean estimation task for other ROIs. A positive though nonsignificant trend was found in other regions including V1, V3, hV4, and IPL (mentioned EVC regions nearly reached significance with *p* = 0.11; Extended Data Fig. 7-1). However, decoding accuracy of V3 and V1 were highly significantly correlated with the decoding accuracy in LOC (vs V1, *ρ* = *0.6*; *p* = 0.001; vs V3, *ρ* = 0.63; *p* < 0.001), indicating a strong association between ensemble-related representations in LOC and early visual regions. Interestingly, the representation and behavioral correlation are stronger in LOC than in low visual areas, perhaps suggesting that one possibility is feedback from LOC to these regions.

In summary, we used MVPA to investigate the neural representation of average orientation in visual-bar ensembles. We found that ensemble mean orientations could be decoded most reliably and robustly from LOC, with converging evidence across multiple analyses. Both ROI-based and searchlight-based decoding analyses pinpointed LOC, with additional support from a complementary correlation-based MVPA. Furthermore, decoding performance in LOC was positively correlated with behavioral performance across participants, supporting functional relevance of LOC. These converging observations imply that ensemble statistics are also included in LOC's well-established role in object-related visual processing (Malach et al., 1995; Grill-Spector et al., 1998; Kourtzi and Kanwisher, 2001; Ayzenberg and Behrmann, 2022a).

## Discussion

To understand this novel result, recall that cognitive studies of object perception have concluded that the visual system establishes “object files, each containing information about a particular object in the scene” (Kahneman et al., 1992). As mentioned, multiple fMRI studies have shown that LOC represents object-related characteristics (Eger et al., 2008; Roth and Zohary, 2015). Furthermore, Ayzenberg and Behrmann (2022a) summarized evidence that ventral LOC may only represent object features, while global object shape information including spatial relations among an object's parts, may be represented primarily in the dorsal pathway (Ayzenberg and Behrmann, 2022b). Thus, LOC may be perfectly suited to represent ensembles, considered as are objects, by their own associated “ensemble file” containing ensemble information such as mean, range, variance, distribution, etc. Our finding of robust LOC representation of ensemble mean orientation confirms this view that the brain represents ensembles as it does objects in terms of their features or characteristics. This is a conceptual novelty in the understanding of how ensembles are represented, as well as what defines an “object” represented in LOC.

The main finding that ensemble average representation was strongest at intermediate-high cortical level LOC is also consistent with RHT (Hochstein and Ahissar, 2002; Ahissar and Hochstein, 2004), which proposes that global ensemble information, such as average orientation, emerges at higher visual levels based on pooled lower-level signals. In this context, ensemble-related information expressed in LOC may reflect access to these global structural properties of the stimulus rather than detailed item-level representations, which are more strongly expressed in EVC. Conscious access to higher-level cortical representations may explain how we perceive averages without explicit knowledge of the local elements.

At the behavioral level, participants were generally able to perform the 2-AFC averaging task with above-chance accuracy, indicating they could extract average orientation information from briefly presented bar arrays (Fig. 3). Importantly, decoding performance in LOC was positively correlated with behavioral performance across participants (Fig. 7), indicating that individuals with more discriminable neural codes for ensemble orientation in LOC tend to perform better in estimating the mean. Consistently, poor behavioral performers also demonstrated the poorest LOC decoding. This relationship further supports the functional relevance of LOC in ensemble representation, suggesting the voxel activity pattern reflects the averaging process required by the task.

Our stimulus design aimed to promote integration across the array rather than reliance on individual-item cues. Ensembles were deliberately constructed with substantial orientation overlap, such that no single bar orientation was diagnostic of any single category. This overlap discouraged item-based strategies and promoted integration across the array—consistent with ensemble perception models. To further minimize item-level encoding, item positions varied unpredictably and were presented briefly (250 ms), limiting focused attention and saccadic selection. While early visual areas may still encode some individual-item information, the overall decoding patterns—particularly in higher-level regions—suggest a representation of global ensemble properties rather than isolated item features.

Extrastriate visual area V3 and parietal cortex areas (SPL and IPS; Figs. 4, 6) also demonstrated ensemble representations, though to a lesser degree. While the largest overlap of searchlight clusters were with bilateral LOC, there was some overlap also in left IPS and SPL and bilateral clusters across EVC regions, especially V3. These findings are consistent with the idea that ensemble statistics are represented bilaterally and in a distributed fashion, spanning multiple levels of the visual hierarchy (Konen and Kastner, 2008; Tark et al., 2021; Robinson et al., 2025). The different areas may have different contributions to ensemble representation, with V3 reflecting residual information about the distribution of orientations or encoding of summary patterns through local feature pooling, while parietal region involvement may reflect higher-order cognitive task-related processes (Wojciulik and Kanwisher, 1999; Peers et al., 2005; Jones and Berryhill, 2012).

An additional aspect tested in this study was the structural representation of mean orientation categories. We observed a gradual decline in prediction probability as the represented orientation diverged from the actual mean, supporting a similarity-based representation. Correlation-based MVPA reinforced this notion, illustrating pattern similarity between adjacent categories, especially in EVC (Fig. 5).

Such similarity patterns are consistent with continuous population codes (Haberman and Whitney, 2012; Hochstein, 2016; Baek and Chong, 2020; Utochkin et al., 2024). In these models, ensemble statistics emerge from pooling responses across neurons tuned to individual items, producing a distribution whose peak reflects the average. Our findings are compatible with this idea, as both distance-based decoding performance and correlation-based MVPA reflect graded similarity between nearby categories.

The neural underpinnings of ensemble perception show considerable variability across studies, possibly reflecting differences in stimuli, task demands, and methodological approaches, as ensemble perception is not a unitary process but supports multiple distinct perceptual goals (Tharmaratnam et al., 2026). Cant and Xu (2012, 2015) employed an adaptation paradigm, defining ensemble selectivity as producing fMRI responses that adapt (as they do for repeated identical scenes) even when ensembles have elements with different local shape features. This was observed in the PPA but not in LOC. On the contrary, LOC responded well to the ensembles, but the responses showed release from adaptation even for different images that shared constituent elements. The difference from our results may derive from their use of ensembles of well-defined objects, so that PPA responded to the ensemble scene and LOC responded to the object elements themselves rather than to the ensemble as a whole. Our use of basic bar stimuli avoided LOC representation of the elements and also avoided scene-like stimuli (explaining absence of PPA response).

In addition, behavioral task differences may underly differences in results: Cant and Xu directed participant attention to the global scene, asking them to report image similarity or change (2012, Experiments 2 and 3) or stimulus aspects unrelated to the ensemble, while we specifically asked participants to explicitly judge ensemble average orientation. Thus, we found an LOC representation of ensemble average, while Cant and Xu, though not finding release from adaptation, nevertheless, found LOC responses to ensemble stimuli (even greater responses than in PPA).

Interestingly, Cant and Xu (2012, 2015) also reported PPA ensemble representation for implicit ensemble averaging, including cases in which they were task-irrelevant or unattended. Similarly, Tark et al. (2021) found that both explicit and implicit orientation ensemble processing engage regions along the hierarchy of EVC, with V3 playing a central role. They found gradual increases in mean orientation-selective responses from V1 to V3 during explicit averaging, while implicit averaging effects were localized primarily to V3. In both studies, parietal regions contributed primarily under explicit task demands. Our classification-based MVPA similarly identified V3 as particularly informative for explicit ensemble orientation representation within EVC (Fig. 4). While we did not include an implicit averaging condition, we observed comparable evidence of explicit ensemble representation in parietal cortex regions (Figs. 4, 6). Further research is needed to test whether LOC also shows a robust implicit representation of ensembles (Khayat and Hochstein, 2018; Hansmann-Roth et al., 2021; Khayat et al., 2024).

Taken together, these studies suggest that visual ensembles are represented in a distributed and dynamic manner, engaging distinct neural networks depending on the content, attentional state, and behavioral relevance. Rather than being localized to a single cortical area, ensemble representation appears to follow a hierarchical model, emerging at multiple levels of the visual system. Depending on perceptual and cognitive demands, it may flexibly recruit ventral or dorsal pathways and incorporate feedback from higher-level regions involved in cognition and memory (Konen and Kastner, 2008; Ayzenberg and Behrmann, 2022a,b).

While decoding shows that ensemble information is present in neural patterns, it cannot determine whether these patterns arise from explicit averaging via an integration mechanism, pooled responses of item-tuned neurons, or subsampling strategies. Nevertheless, the robust correlation with behavioral ensemble mean estimation indicates that LOC representations are not incidental but functionally linked to perceptual encoding of ensemble summaries. This brain–behavior relationship strengthens the interpretation that decoding reflects meaningful summary representations, even if the precise underlying mechanism remains unresolved.

Although using oriented bars ensured precise control, the current design limits generalization to more complex or naturalistic ensembles. Future studies could extend this work by testing other ensemble dimensions of higher-level object features, manipulating spatial structure and temporal presentation, and further investigating how ensemble representations emerge automatically or are modulated by top–down attention and task relevance. The role of LOC should also be tested for implicit ensemble perception. Additionally, details of how LOC represents “object files,” for objects and for ensembles, need further investigation.

## Conclusion

Our findings suggest that ensemble mean orientation may be represented predominantly in object-related LOC, presumably as part of a distributed cortical network that may also include early visual areas and parietal cortex. LOC representation was supported by converging evidence across multiple analyses. The behavior-decoding correlation indicates a functional link between ensemble average representation in LOC and perceptual accuracy. In addition, we found graded decoding and correlation-based similarity patterns, which point to orientation-structured neural representations. Whether these representations arise from population pooling mechanisms, midlevel grouping processes, or task-dependent computations remains to be determined. Taken together, our results provide a foundation for further investigation into how the visual system summarizes information across space and encodes ensemble statistics. Ensemble properties, such as mean, range, variance, distribution, etc., may be seen as contained in an “ensemble file,” classically including object properties, leading to ensembles also being represented in object-area LOC. The implications for how objects are represented in LOC also require further study.

## Synthesis

Reviewing Editor: Niko Busch, Westfalische Wilhelms-Universitat Munster Decisions are customarily a result of the Reviewing Editor and the peer reviewers coming together and discussing their recommendations until a consensus is reached. When revisions are invited, a fact-based synthesis statement explaining their decision and outlining what is needed to prepare a revision will be listed below. The following reviewer(s) agreed to reveal their identity: NONE. Note: If this manuscript was transferred from JNeurosci and a decision was made to accept the manuscript without peer review, a synthesis may not be available.

# Synthesis

This manuscript has been transferred from the Journal of Neuroscience, where it was reviewed by two experts on object perception and ensemble statistics. The authors have revised the manuscript in response to the reviewers' comments. Having evaluated the reviews and the authors' responses and revisions, I am making the decision to accept this paper for publication in eNeuro. The reviews were generally positive, and the revisions provided by the authors -- especially those concerning the novel theoretical contribution of this study -- are adequate.

I only have one minor remark for the authors: your manuscript currently includes several supplemental figures and tables. Please note that eNeuro does not use supplemental material. Please either submit this content as "Extended Data" or, preferably, incorporate it into the main text.
