# Supplementary material for: Neural Patterns Reveal Lateral Occipital Complex Representation of Ensemble Mean Orientation
Source: eNeuro. 2026 Jun 30;13(7):ENEURO.0137-26.2026. doi: 10.1523/ENEURO.0137-26.2026 (PMC13338491; doi:10.1523/ENEURO.0137-26.2026)
Supplement: Figure 2-1 — MNI coordinates per ROI. Details of ROI coordinates (center of mass). Download Figure 2-1, DOCX file. [file eneuro-13-ENEURO.0137-26.2026-s006.docx]

**Extended Data Fig 2-1. MNI coordinates per ROI**

| **ROI** | **Center of Mass (L)** | | | | | **Center of Mass (R)** | | |
| --- | --- | --- | --- | --- | --- | --- | --- | --- |
|  | | **x** | **y** | **z** | **x** | | **y** | **z** |
| V1 | | -7.3 | 87.4 | 5.2 | 8.6 | | -82.3 | 8.5 |
| V2 | | -8.5 | -90 | 4 | 9.4 | | -86 | 8.6 |
| V3 | | -13.6 | -90 | 3.9 | 14.1 | | -86.2 | 8.3 |
| hV4 | | -20.5 | -87.3 | -13.6 | 18.7 | | -85.3 | -10.4 |
| FFA | | -42 | -54.4 | -20.1 | 42.2 | | -51.7 | -20.1 |
| LOC | | -40.4 | -76.1 | -0.5 | 40.1 | | -77.7 | 2.5 |
| PPA | | -25.5 | -45.5 | -10.2 | 26.4 | | -42.9 | -10.9 |
| IPL | | -47 | -44.1 | 42.6 | 48.1 | | -44.5 | 43 |
| SPL | | -26.2 | -64.3 | 53.3 | 27.6 | | -64.2 | 53.5 |
| TPJ | | -49.8 | -67.5 | 22.8 | 52.5 | | -67.5 | 22.2 |

MNI x, y, z coordinates representing the center of mass for each region of interest (ROI), defined across all participants.
