# Supplementary material for: Neural Patterns Reveal Lateral Occipital Complex Representation of Ensemble Mean Orientation
Source: eNeuro. 2026 Jun 30;13(7):ENEURO.0137-26.2026. doi: 10.1523/ENEURO.0137-26.2026 (PMC13338491; doi:10.1523/ENEURO.0137-26.2026)
Supplement: Figure 2-2 — ROI size (voxel count). Details of ROI size (group mean ± SD) Download Figure 2-2, DOCX file. [file eneuro-13-ENEURO.0137-26.2026-s007.docx]

**Extended Data Fig. 2-2. ROI size (voxel count)**

| **Bilateral ROI** | **mean** | **SD** |
| --- | --- | --- |
| V1 | 1333.4 | 274.1 |
| V2 | 1057.3 | 243.1 |
| V3 | 1143.5 | 429.5 |
| hV4 | 269.2 | 97.1 |
| FFA | 286.5 | 133.7 |
| LOC | 1325.3 | 736.5 |
| PPA | 263.8 | 79.3 |
| IPL | 682 | - |
| SPL | 407 | - |
| TPJ | 787 | - |

Mean and standard deviation of the number of voxels included in each ROI.
