# Supplementary figures and images for: Neural Patterns Reveal Lateral Occipital Complex Representation of Ensemble Mean Orientation
Source: eNeuro. 2026 Jun 30;13(7):ENEURO.0137-26.2026. doi: 10.1523/ENEURO.0137-26.2026 (PMC13338491; doi:10.1523/ENEURO.0137-26.2026)

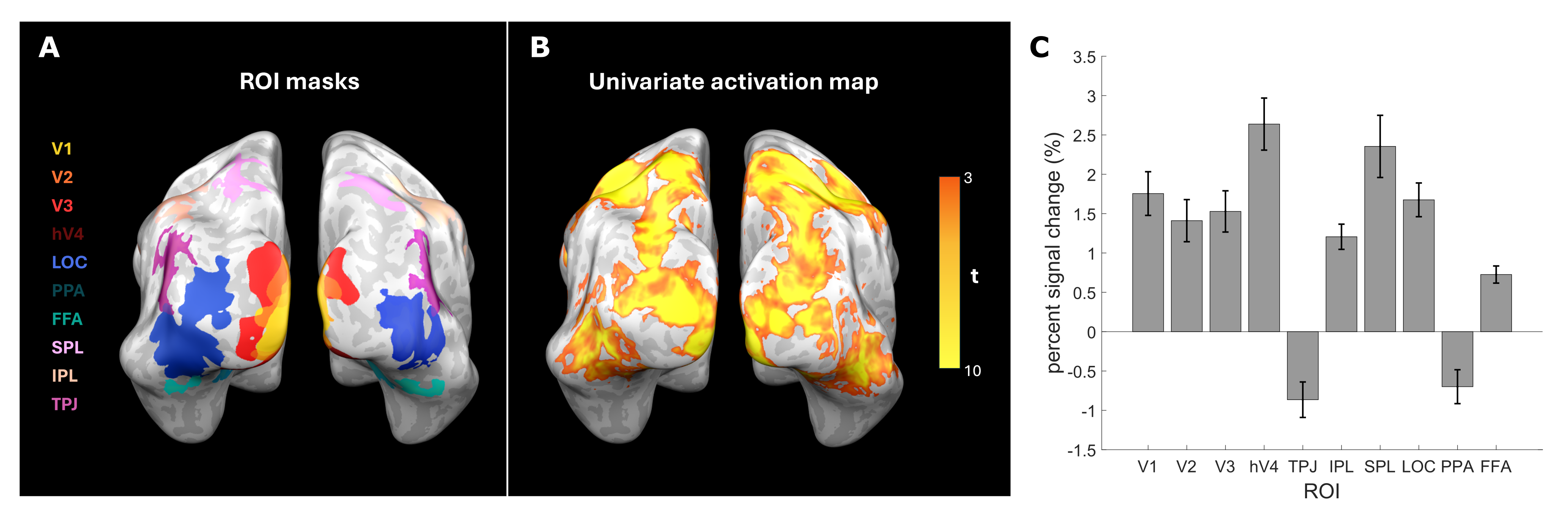

Supplement: Figure 4-1 — Group-level ROIs and univariate activation analyses. (A) Group-level ROIs thresholded at ≥20% participant overlap. (B) group activation map illustrating overall activation for bar stimuli across all orientations. (C) Univariate analysis of mean percent signal change relative to fixation for each ROI. Overall activation was generally positive, but did not predict decoding performance, which relies on condition-specific spatial activation patterns rather than average signal strength. Bars represent group means; error bars indicate ± SEM. Download Figure 4-1, TIF file. [file eneuro-13-ENEURO.0137-26.2026-s005.tif]

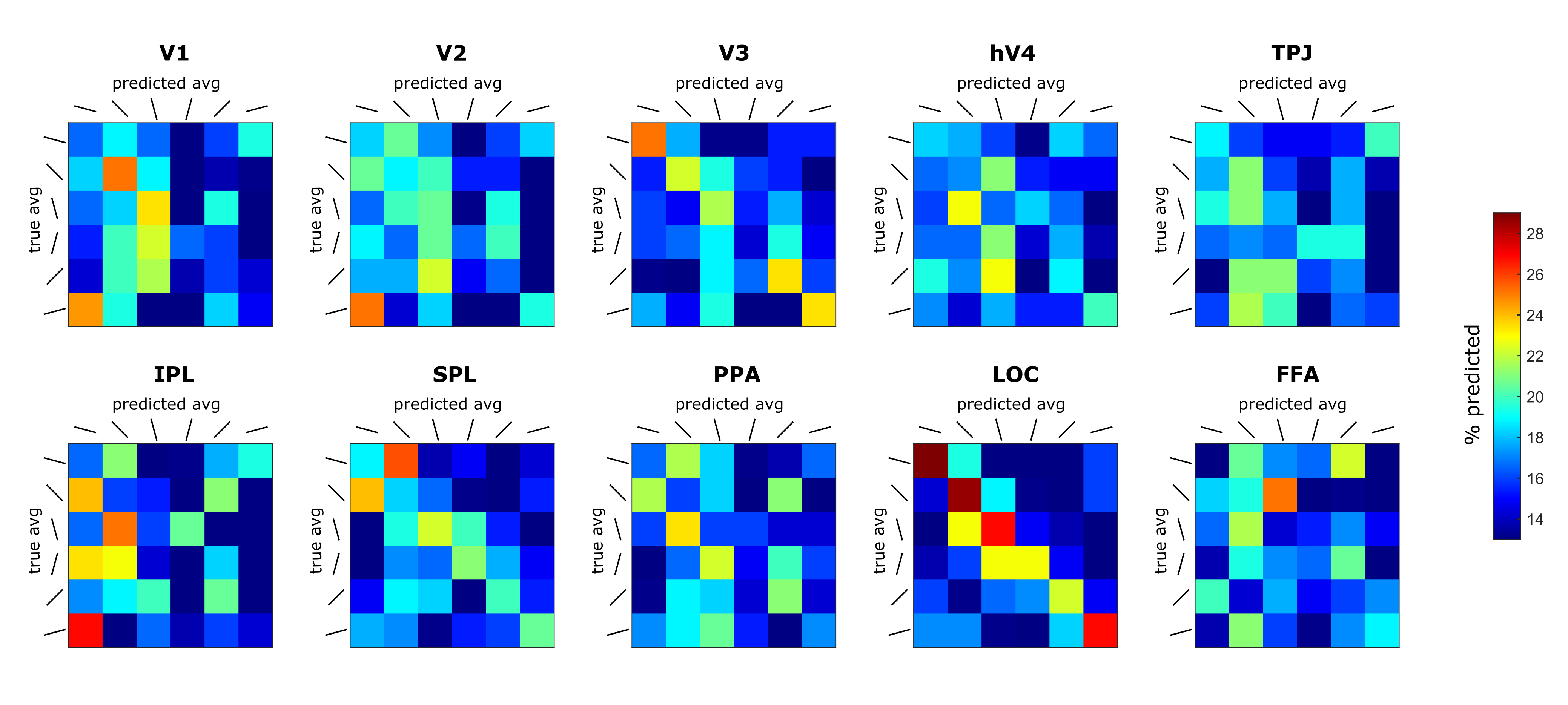

Supplement: Figure 4-2 — Confusion matrices of classification accuracy for all ROIs. Confusion matrices showing classification accuracy across all ensemble orientation mean categories. Values are averaged across participants. ROIs with significant classification accuracy are reported in the main text (Fig. 4B). Download Figure 4-2, TIF file. [file eneuro-13-ENEURO.0137-26.2026-s004.tif]

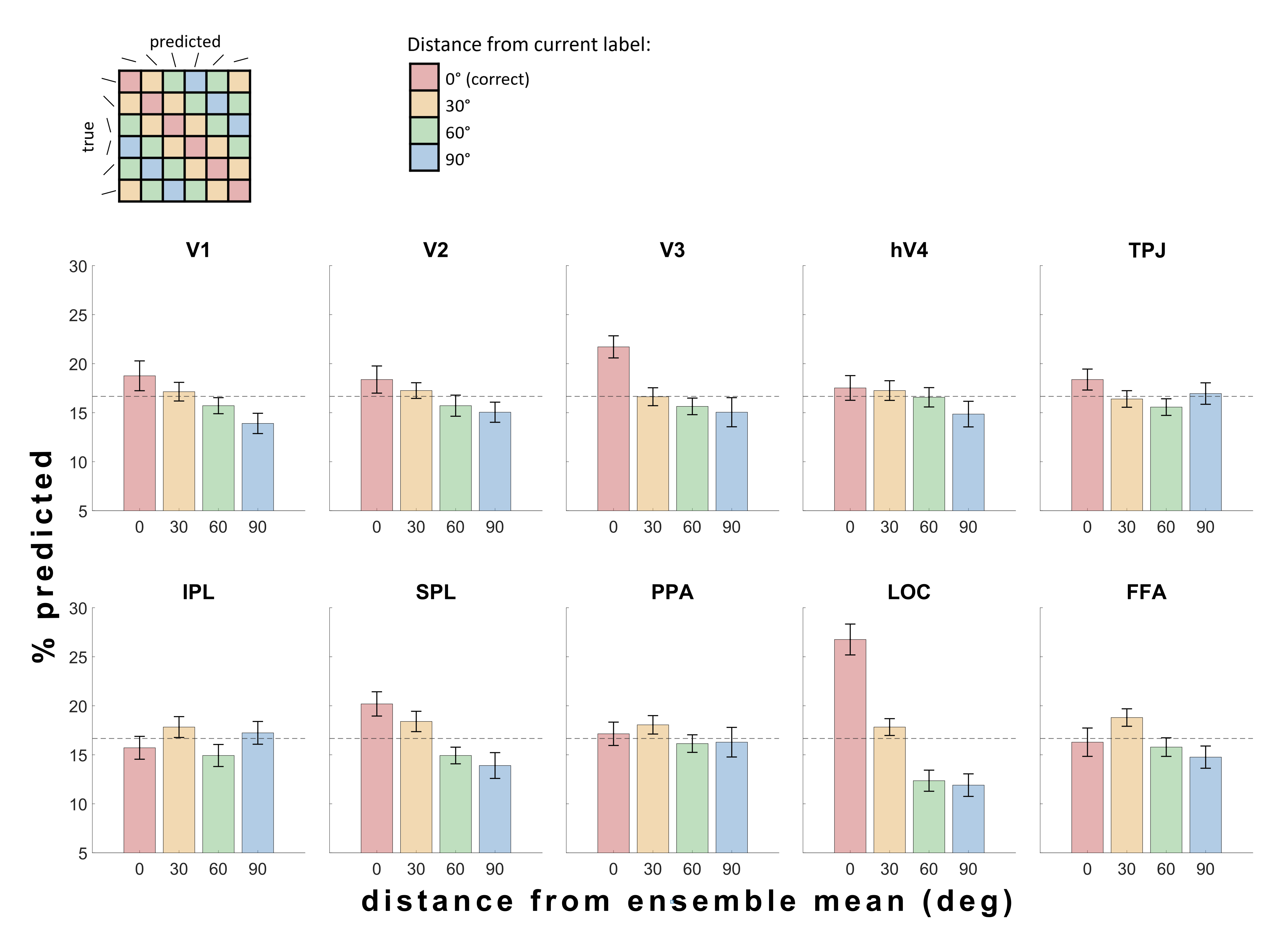

Supplement: Figure 4-3 — Percent of prediction as a function of angular distance from the true ensemble mean for all ROIs. Top: Analysis scheme illustrating how confusion matrix entries were grouped by orientation difference. Bottom: Mean prediction rate at each distance, across ROIs. Bar colors correspond to distance from the true mean. Gradient decrease indicates similarity representation by decoding values that vary with distance from the actual mean orientation in several ROIs. Download Figure 4-3, TIF file. [file eneuro-13-ENEURO.0137-26.2026-s003.tif]

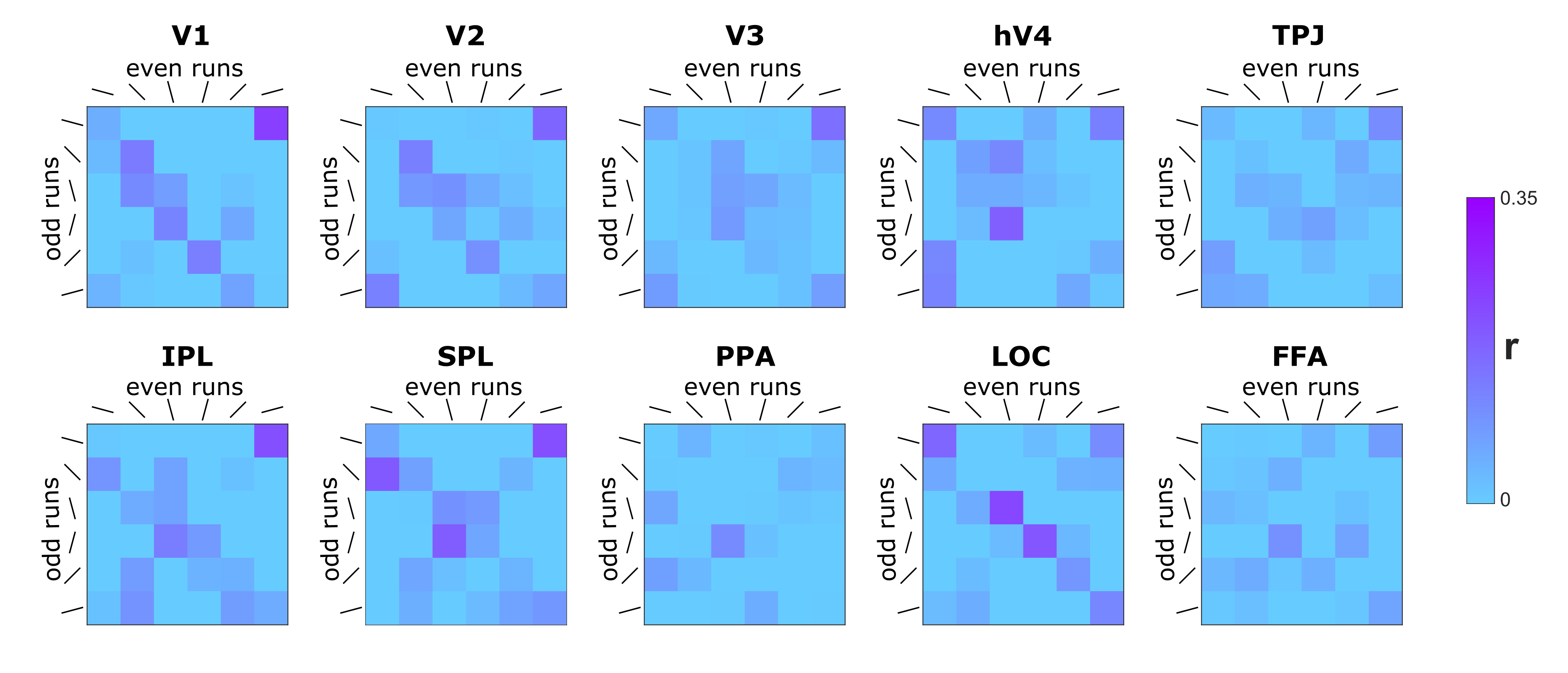

Supplement: Figure 5-1 — Correlation-based MVPA confusion matrices for all ROIs. Correlation matrices between odd and even runs for each pair of mean orientation categories, displayed separately for all 10 ROIs. Correlations along the main diagonal reflect within-category similarity, whereas off-diagonal bands indicate similarity between adjacent categories. Download Figure 5-1, TIF file. [file eneuro-13-ENEURO.0137-26.2026-s002.tif]

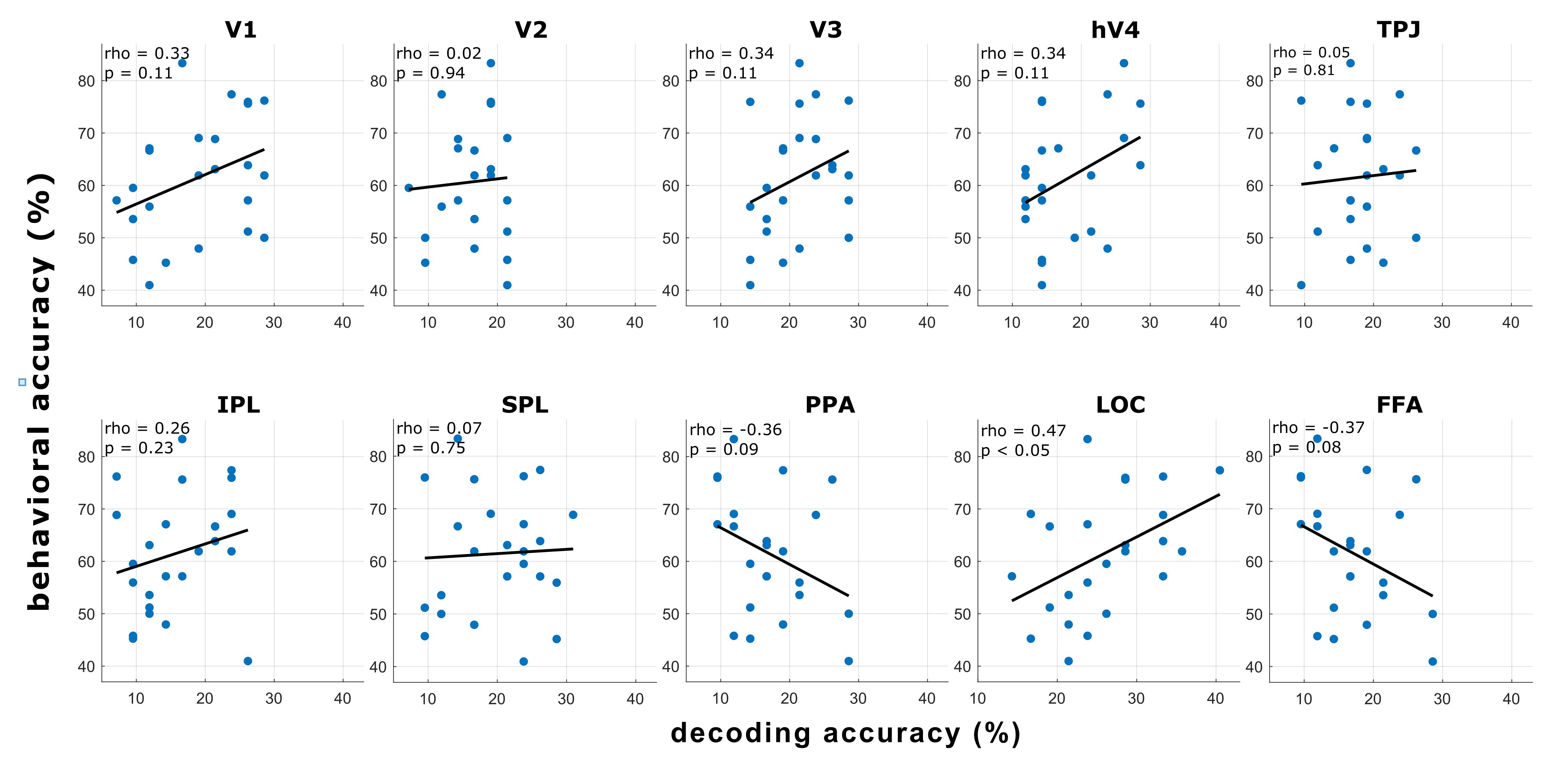

Supplement: Figure 7-1 — Behavioral–neural correlations for all ROIs. Correlations between individual decoding accuracy and behavioral catch-trial accuracy across ROIs. Although some positive trends are observed, none reach Spearman correlation significance besides LOC, illustrating a relationship of the inter-individual variability in neural decoding and perceptual averaging behavioral. Each dot represents one participant included after exclusion of 2 extreme outliers (±2SD criterion applied to both behavioral and MVPA data), and the fitted regression line illustrates the linear slope. Download Figure 7-1, TIF file. [file eneuro-13-ENEURO.0137-26.2026-s001.tif]
