# Supplementary material for: Neural Patterns Reveal Lateral Occipital Complex Representation of Ensemble Mean Orientation
Source: eNeuro. 2026 Jun 30;13(7):ENEURO.0137-26.2026. doi: 10.1523/ENEURO.0137-26.2026 (PMC13338491; doi:10.1523/ENEURO.0137-26.2026)
Supplement: Figure 5-2 — Correlation analysis of orientation ensemble mean. Full statistical correlation analysis results. Download Figure 5-2, DOCX file. [file eneuro-13-ENEURO.0137-26.2026-s008.docx]

**Extended Data Fig. 5-2. Correlation analysis of orientation ensemble mean**

|  | **ROI** | **two tailed *t*-test** | | |
| --- | --- | --- | --- | --- |
|  |  | ***t*** | ***p*** | ***p-corrected*** |
| Within-between category pattern correlation | V1 | 1.225 | 0.232 | 0.928 |
|  | V2 | 2.005 | 0.056 | 0.439 |
|  | V3 | 1.776 | 0.088 | 0.603 |
|  | hV4 | 1.676 | 0.106 | 0.675 |
|  | LOC | 5.785 | 5.79 × 10^-6^ | 5.79 × 10^-5^ |
|  | PPA | 0.283 | 0.779 | 0.999 |
|  | FFA | -0.179 | 0.859 | 0.999 |
|  | IPL | 1.771 | 0.089 | 0.606 |
|  | SPL | 2.746 | 0.011 | 0.106 |
|  | TPJ | 1.249 | 0.223 | 0.920 |

Statistical results per ROI across 25 participants. Reported are differences between within-category and between-category pattern correlations. For each ROI, within- vs. between-category correlation differences were evaluated using one sample two-tailed *t*-tests. *p*-values were corrected for multiple comparisons using Sidák correction.
